# Preparation and Characterization of Carbon Nanodots from turmeric soot for anti-coliform and anti-oral bacterial applications and as anti-staphylococcal coatings

**DOI:** 10.7150/ntno.99825

**Published:** 2025-01-01

**Authors:** Arjun Prakash, Manikandan Muthu, Gnanadeepam Raja, Judy Gopal

**Affiliations:** 1 Saveetha Medical College, Saveetha Institute of Medical and Technical Sciences (SIMATS), Thandalam, Chennai - 602105, Tamil Nadu, India.; 2 Department of Research and Innovation, Saveetha School of Engineering, Saveetha Institute of Medical and Technical Sciences (SIMATS), Thandalam, Chennai - 602105, Tamil Nadu, India.

**Keywords:** smoke, carbon nanodots, coliforms, antibiofilm, oral bacteria

## Abstract

In an era where chemical synthesis of nanomaterial is accounting for the generation of toxic wastes, leading to nanotoxicity, the present work focuses on the extraction of carbon nanodots from available natural sources such as turmeric smoke. The extracted carbon nanodots were characterized and their physical and chemical attributes were confirmed. The antibacterial property of the isolated carbon nanodots was tested against coliforms and oral bacteria. The results indicated that the carbon nanodots possessed highly versatile antibacterial activity. Carbon coatings were prepared by dip-dry method from the turmeric smoke-derived nanodots. The carbon-coated glass surfaces showed biofilm-repellant activity when exposed to pathogenic Staphylococcal cultures. The bactericidal and antibacterial activity of the in-house extracted carbon nanodots was proved. This study introduces an ecofriendly and simple rapid carbon nanomaterial synthesis process from smoke which can be extended for various other applications too.

## 1. Introduction

The complex strands that make up nature's floral tapestry frequently hide amazing qualities. Among these, turmeric—a member of the Zingiberaceae family of ginger and formally known as *Curcuma longa*—stands out as a symbol of its diverse range of therapeutic applications 1. Admired for many years in Asian nations, this blooming flowering plant has become more important for its role in traditional medicine than it has in the culinary world 2. Turmeric's rhizomes, which are prized for their fragrant essence, contain an advantageous wealth of beneficial properties, including strong antiseptic, anti-inflammatory, and disinfection properties 3.

Turmeric has a long history of use in medicinal practices. Burning Turmeric is a customary traditional practice that has been used to treat a wide variety of illnesses, from colds to immune system strengthening. Its function in fostering healthy good gut flora, correcting the LDL cholesterol balance, and improving skin tone has been discussed 4.

There are 6.3% proteins, 69.4% carbohydrates, 5.1% lipids, 13.1% water, 3.5% minerals, and 5% curcuminoids in turmeric. Three of the compounds that are referred to as curcuminoids are desmethoxycurcumin, bisdemethoxycurcumin, and curcumin (diferuloyl methane). The yellow hue and majority of turmeric's health benefits are attributed to curcumin, which comprises 3 to 4% of the spice 5, 6.

Turmeric possesses remarkable properties that stem from curcumin, one of its main constituents, including anti-inflammatory and antioxidant properties 7. Scientific studies have confirmed these characteristics, adding reliance to the plant's medicinal properties. Sinusitis, common colds, and immune system support are just a few of the many medical issues that turmeric can help with 7. As such, the many health benefits of turmeric have earned it great esteem in traditional medicine systems. Curcumin has multiple properties, including as an antibacterial and a wound-healing agent 8. Furthermore, turmeric has medical, cosmetic, and culinary uses. There is an age-old traditional household practise in Asian countries, where the smoke from burning turmeric sticks when inhaled, spontaneously clears nasal congestion.

The objective of the present study is to extract the carbon nanodots from the turmeric smoke and characterize the nanomaterial to disclose its identity. The primary aim of isolating this nanomaterial is also to produce an environmentally acceptable nanomaterial that possesses bactericidal properties against oral bacteria and coliforms such *Enterobacter faecalis* and *E. coli*. We also assessed these coatings' ability to inhibit the growth of biofilms, using the carbon nanodots produced from turmeric smoke.

## Materials and Methods

### Collection of turmeric soot

After purchasing some turmeric rhizome sticks at a nearby grocery, they were exposed to the flame of a Bunsen burner which were burned and once the rhizome became red hot, it was taken away from the flame. The smoke emitted from the burning rhizomes was gathered into a glass beaker. This process was repeated numerous times in order to amass high concentrations of turmeric soot.

The turmeric soot accumulated in the beaker was removed from the inner surfaces of the beaker by subjecting it to ultrasonication (BOROSIL UCB050 5 Ltr Ultrasonic Bath 120 W) for 10 minutes in 50 millilitres of sterile water. The carbon material on the beaker walls were dislodged and was evenly distributed in water after putting it through ultrasonication. Next, the carbon nanodots were extracted from the solution by subjecting the mixture to centrifugation at a speed of 10,000 rpm for 10 minutes 9. The carbon nanodots were found in the supernatant, which was employed for additional experimentation while the brownish black precipitate was disposed of.

### Characterization of the carbon nanodots

A Scanning Electron Microscope (JSM -IT800 NANO SEM) verified the morphology, shape and size distribution of the carbon nanodots. A UV-visible spectrophotometer (Thermo Fisher Scientific, NanoDrop Lite Plus UV) was utilized to analyze the optical properties and absorbance of the carbon nanodots. A Fourier-transform infrared spectroscopy (FTIR) (Bruker, Compact FT-IR Spectrometer ALPHA II) was used to characterize and examine the functional groups and chemical composition of the carbon nanodots.

### Anti-coliform assay

**Figure 1:**
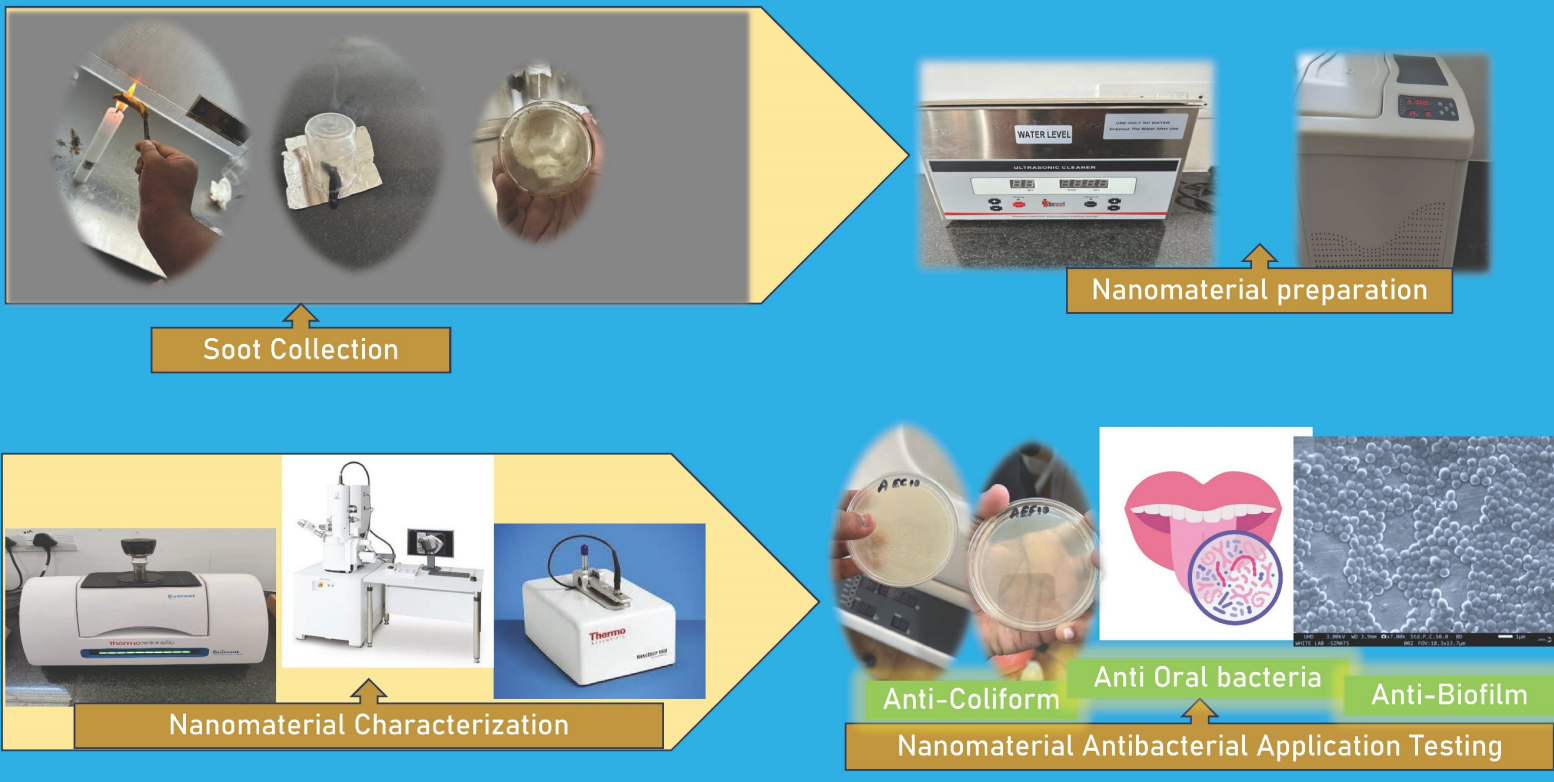
Scheme showing work flow involved in the study.

The efficiency of turmeric smoke derived carbon nanodots against coliform bacteria, specifically against *E. coli* and *E. faecalis*, was determined. *E. coli* and *E. faecalis* were exposed to different concentrations of carbon nanoparticles generated from turmeric soot during incubation. The bacterial strains used in the study were obtained from the culture collection of Department of Microbiology, Centre for Infectious diseases, Saveetha Institute of Medical and technical Sciences (SIMATS), India. The specimens were cultivated in nutritious broth at 37º C, overnight. The quantity of coliform bacteria that survived the interaction with turmeric smoke derived carbon nanoparticles was determined using the plate count method, which involved enumerating the total number of viable bacteria by plating on agar. The TVC was denoted as cfu/mL (colony forming unit/mL) 10. The results, which were obtained from three different trials, are presented as the average value plus or minus the standard deviation. Version 12.0 of IBM SPSS for Windows was used to do the statistical analysis (SPSS Inc., Chicago, IL, USA). Duncan's multiple range test was used to determine the statistical significance of multiple comparisons using a one-way analysis of variance (ANOVA). A p-value below 0.05, 0.01, and 0.001, respectively, was deemed to be statistically significant. Figure 1 presents the schematic workflow employed in the current investigation.

### Anti-oral bacterial assay

The extracted carbon dots' capacity to combat oral microorganisms was evaluated. Oral bacteria samples were gathered from a human volunteer. The bacterial samples were collected using a sterile brush, Then the bacterial suspension was suspended in 20 millilitres of phosphate buffer and kept in falcon tubes in the refrigerator. 500 uL/L of nanomaterial was incubated with the oral samples in triplicates. After the carbon nanomaterial interaction overnight in a shaker cum incubator, the remaining bacteria were counted by plating them on Mitis Salivarius Agar Base (HiMedia M259), employing the TVC technique. The colonies were counted after an incubation period of 48hrs, and the total viable bacteria were calculated using the standard microbiological formulae.

### Anti-biofilm assay

Nanocoatings of carbon were made on glass surfaces by immersing them into the carbon nanodot solution for 24 h. The glass slides were removed and dried in a hot air oven overnight. Glass slides covered with carbon nanodots were submerged in *Staphylococcus aureus* nutrient broth cultures for biofilm formation. The biofilm components that were loosely attached to the slides were removed by retrieving them and gently washing them in sterile water. 0.1% acridine orange, fluorescent dye was flooded onto the biofilms and incubated for 2 min. Following a thorough wash, the slides were allowed to air dry. An Olympus CKX53 Fluorescence Microscope was used to capture images of the biofilm on the coated and unprotected glass surfaces. Acridine orange, is a fluorescent dye, that fluoresces orange when intercalated with single stranded RNA. The number of orange fluorescing cells represent the actively metabolizing cells on the surface 11. ImageJ (Version 1.45k) software was used to quantify the fluorescing cells. Higher fluorescence is an indication of more living cells. The figures presented here are an average of the five locations on the same surface that were scanned using three samples. Figure 1 presents the schematic workflow followed in this paper.

## Results

### Characterization of turmeric carbon nanodots

**Figure 2:**
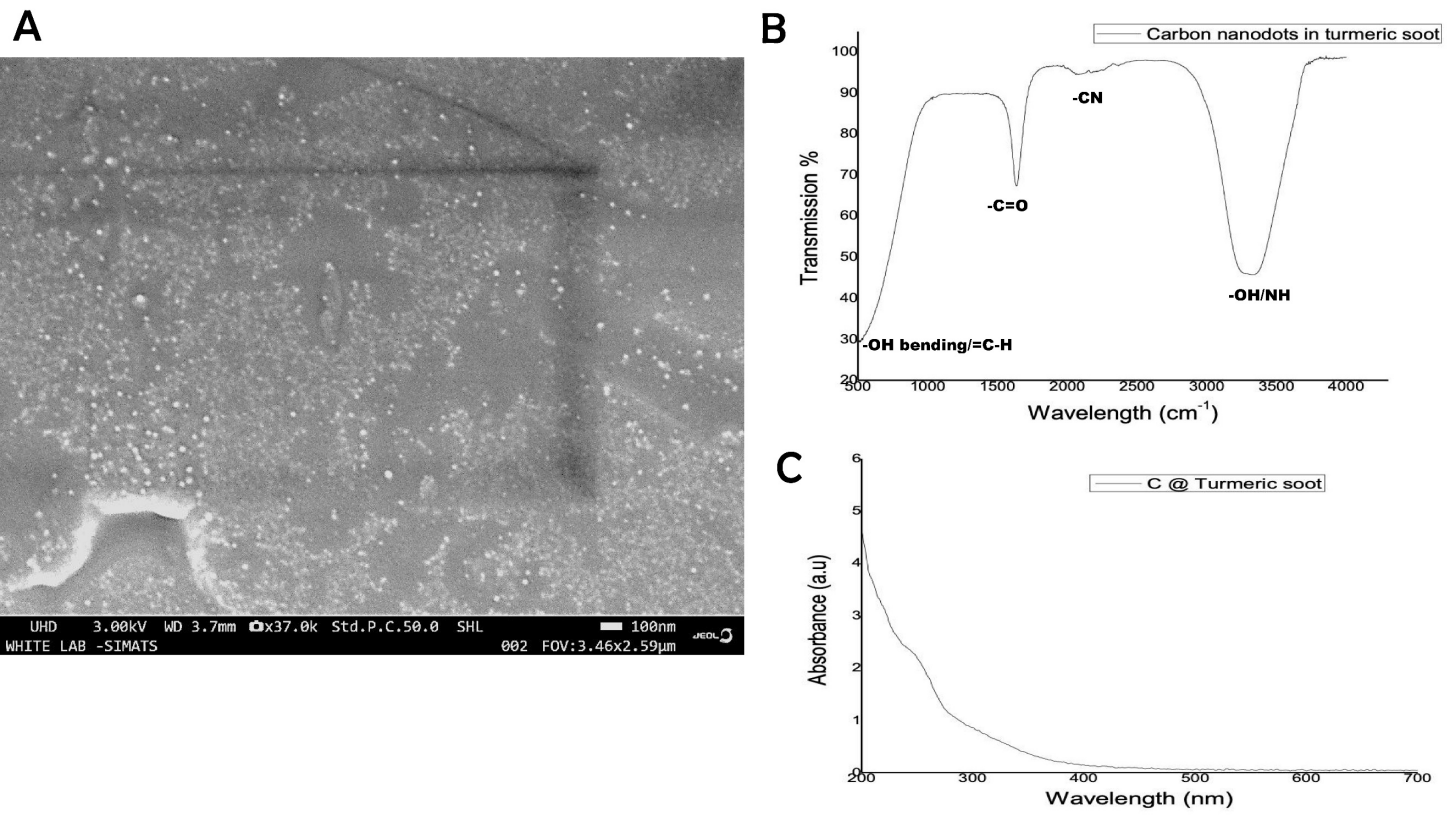
A. NanoSEM micrographs of the turmeric smoke derived carbon nanodots; B. FTIR spectra and (2C) UV-Vis Spectra of the carbon nanodots.

NanoSEM was used to verify the size and shape and the morphology of the carbon nanodots produced from turmeric smoke. Carbon nanodots measuring 12-23 nm were recovered from the turmeric smoke, as shown in Figure 2 SEM micrographs. Previous 9, 11,12 authors have also documented the successful extraction of carbon nanodots from smoke, soot as well as turmeric smoke. The carbon nanodots had irregular particle sizes, which is reported to be similar in case of naturally occurring nanoparticles, especially in soot samples as reported by few other authors too 13,14.

For the purpose of determining the chemical makeup of the nanodots, FTIR and UV-Vis Spectrophotometry were used to characterize the isolated nano carbon. FTIR absorption bands of υ (O-H), υ (C-H) and υ (C = O) at 3320 cm^-1^, 2934 cm^-1^ and 1764 cm^-1^, respectively, confirm the presence of -COOH, which are characteristic of carbon nanoparticles (Fig 2B). For carbon nanoparticles as well, the UV absorption peak ranges from 206 to 280 nm (Fig. 2C). Earlier reports too confirm the FTIR and UV-Vis results obtained in the above study 15.

**Figure 3:**
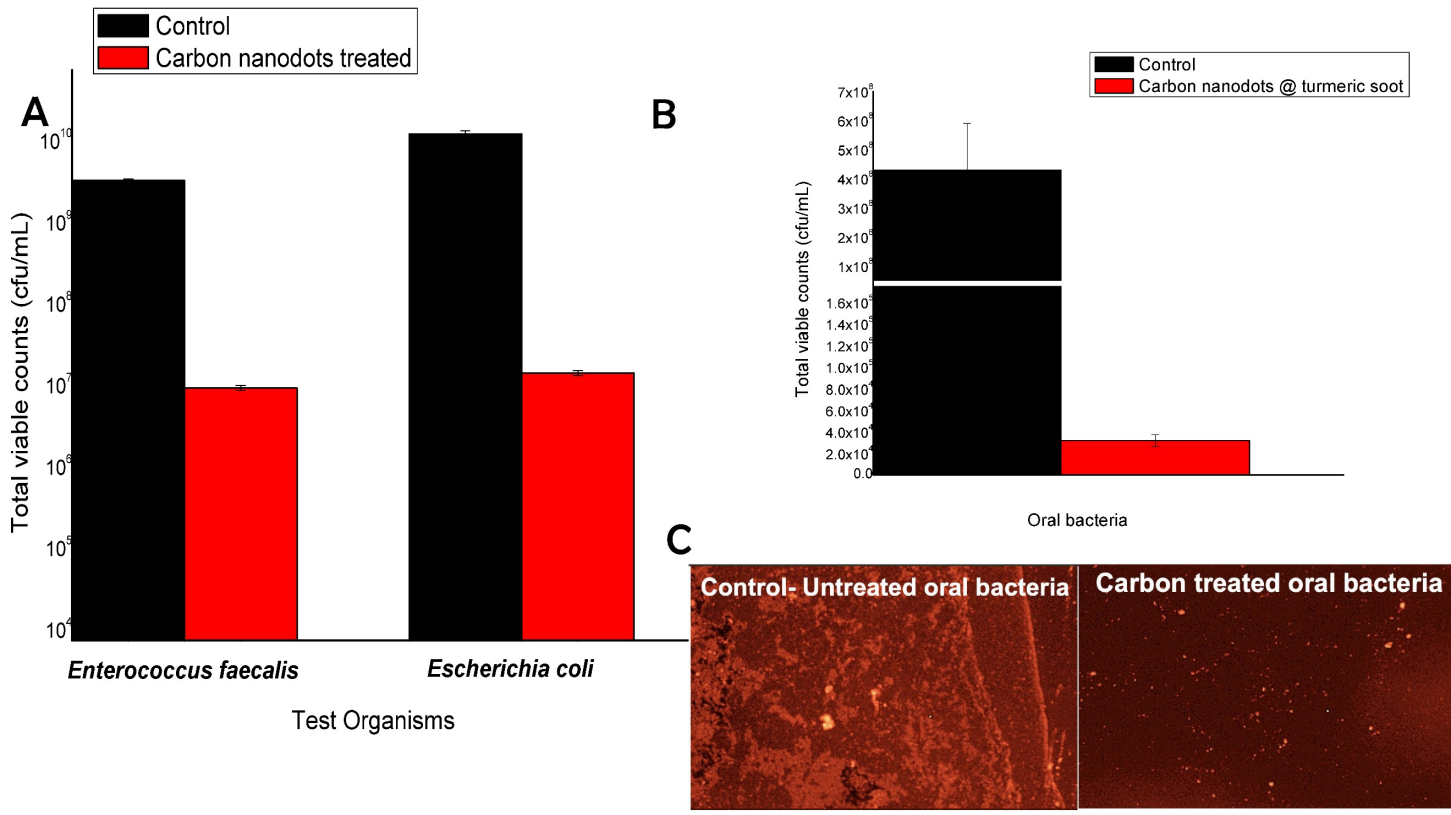
A. Graph showing results of Anti- Coliform activity of turmeric smoke-derived carbon nanodots. Inset shows the distinct inhibition of viable colonies on agar plates owing to the carbon nanodot interaction. * Significant; ** highly significant based on statistical analysis using paired t-tests. B. Results of TVC counts showing inhibition of oral bacteria following incubation with carbon nanodots and (3C) Epifluorescence images showing fluorescing live oral bacteria post-interaction with turmeric smoke-derived carbon nanodots.

The anti-coliform properties of the carbon nanodots produced from turmeric smoke were confirmed by antimicrobial tests. The plate count method's results are shown in Fig. 3A, where statistically highly substantial inhibition of the coliform bacteria *E. faecalis* was observed, while *E. coli* inhibition rates were relatively lesser than that of *E. faecalis*, although it was still deemed significant enough to be considered effective.

The antibacterial activity of the carbon nanodots produced from smoke was demonstrated by the outcomes of the anti-oral bacterial tests. As observed from Fig. 3B, significant inhibition of oral bacteria was observed, confirming the anti-oral bacterial activity of the nanodots.

Figure 3C displays the epifluorescence images of the untreated oral bacteria, showing high fluorescence compared to the treated oral bacteria, that are poorly stained showing low orange florescence.

**Figure 4:**
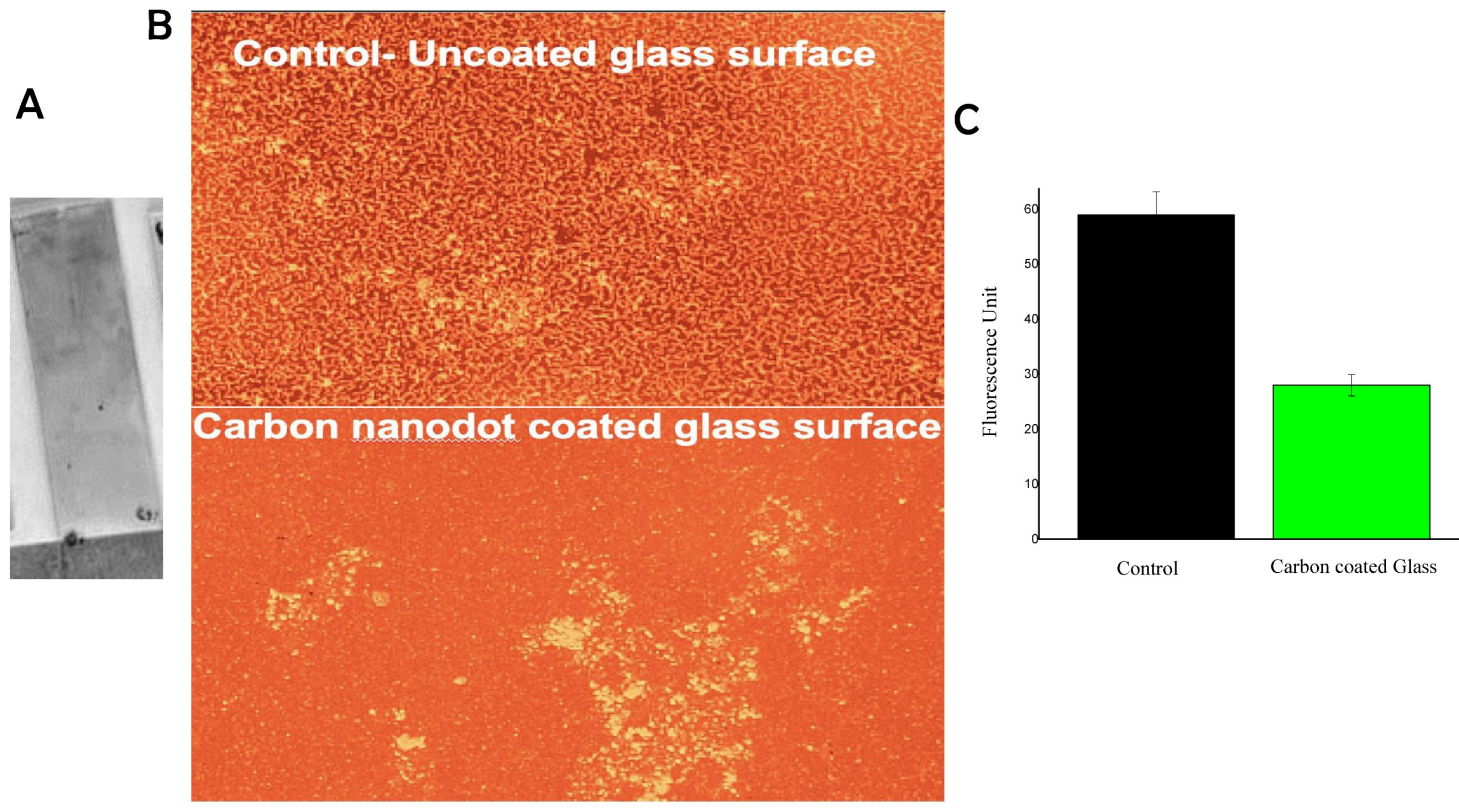
(A) Photograph showing carbon coating on glass. Epifluorescence images of biofilm on (4B) carbon-coated and control glass surfaces and (4C) Graph showing results of quantification of live fluorescing biofilm cells based on ImageJ analysis.

The biofilm on the carbon coatings prepared though dip coating in turmeric soot derived carbon nanodots was assayed. Figure 4A shows the nature of the carbon coating on the glass slide. The antibiofilm capacity of the carbon coated surfaces was validated by the epifluorescence imaging of the bacterial culture exposed surfaces, as shown in Fig. 4B. The biofilm was quantified using image analysis and these results also confirmed significant inhibition of biofilm formation on the carbon coated surfaces compared to the control surface (Fig. 4C).

## Discussion

**Figure 5:**
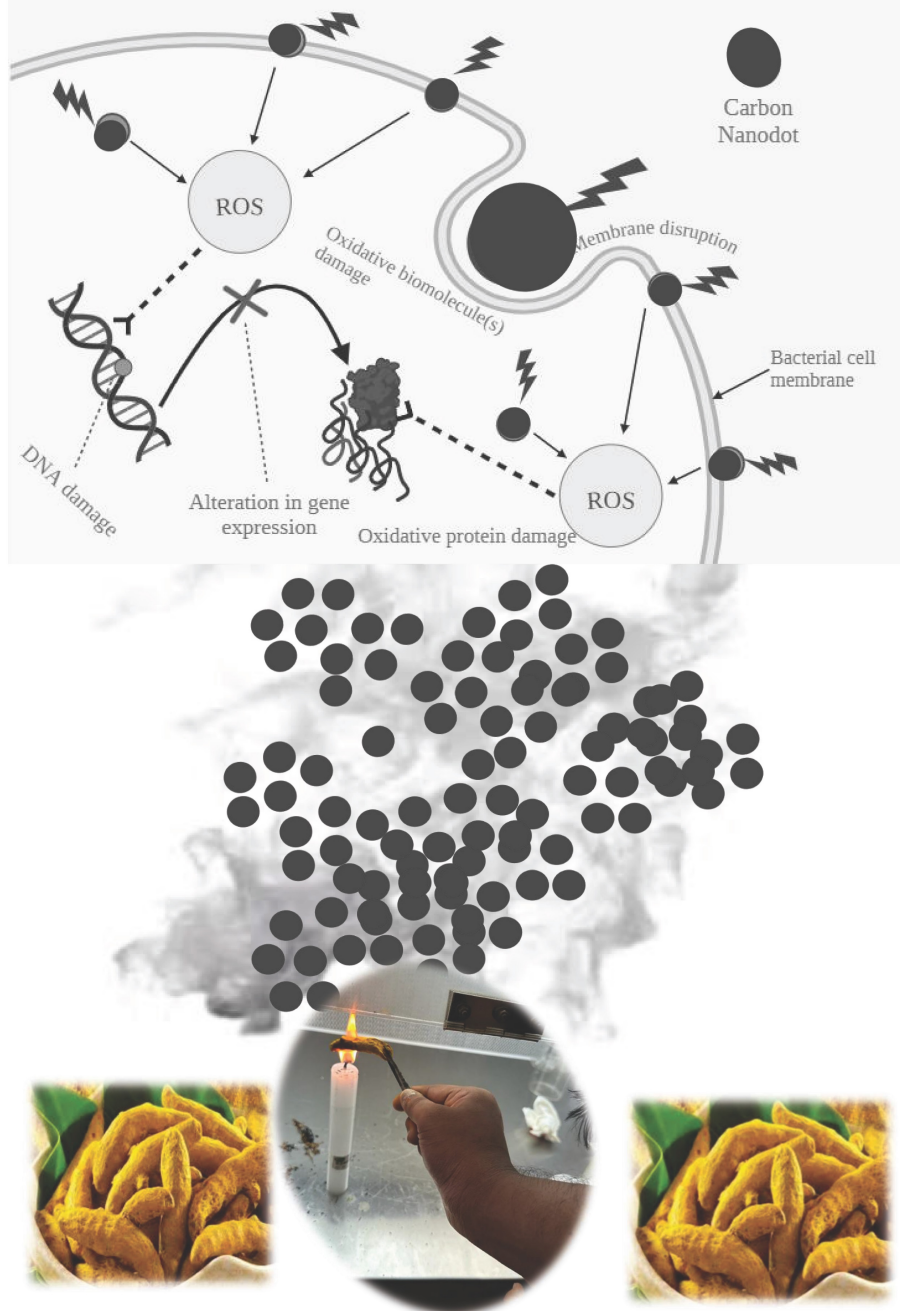
Schematic speculation of the mechanism behind the antibacterial activity of the turmeric smoke derived CNDs.

This study's findings showed that carbon nanomaterials made from turmeric smoke have potent antibacterial properties against coliform bacteria, especially *E. faecalis*. The findings also confirmed that the smoke-derived carbon nanomaterials could result in inhibition of oral bacteria. Additionally, the carbon coatings developed from the carbon derived from turmeric smoke also exhibited strong antibiofilm activity. These findings support the carbon compounds' antibacterial properties that were isolated from turmeric smoke. To pinpoint the particular mechanisms by which these CNDs function as antibacterial agents, further investigation is required. However, it is likely that the distinctive physicochemical characteristics of the CNDs play a major role in rupturing bacterial cell membranes and interfering with crucial cellular functions. Based on the previous reports, we present a schematic diagram of the plausible mechanism behind the antibacterial activity of carbon nanodots (Fig. 5).

These results point out the possible use of carbon nanomaterials from the smoke of turmeric as powerful antimicrobials. Further investigation is necessary to clarify the specific antibacterial mechanisms, optimise the synthesis process, and evaluate the performance of these CNDs in real-world scenarios such as water treatment or biomedical applications. Previous studies 1,2 have documented the antimicrobial properties of medicinal smoke 16. Medicinal smoke reduces airborne bacteria 17. Chun *et al.*, have reported the antibacterial activity of turmeric smoke-derived carbon nanodots 15.

The antibacterial activities of turmeric smoke generated carbon nanodots are also reported to be enabled through functionalization of the carbon nanodots with the bioactive components present in turmeric. The largest bioactive component of turmeric is curcumin (70-75%), a phenolic molecule that is part of the curcumin family and accounts for 2-9% of the total composition 10,11 followed by two compounds that are desmethoxycurcumin (10-25%) and bisdemethoxycurcumin (5-10%). Turmeric also contains 1-(4-hydroxy-3, 5-dimethylethoxyphenyl)-(1E, 6E)-1, 6-heptadiene-3, and other related phenolic compounds 1-hydroxy-1, 7-bis (4-hydroxy-3-methoxyphenyl)-(6E)-6-heptene3, 5-dione-1,4,6 heptatrien-3-one. These bioactive compounds can break into bacterial cells, and once inside the cell, the phenolic chemicals can enter the bacteria and alter metabolism of the bacteria. Turmeric also contains acidic polysaccharides 16-19 and tannins, phytic acid, saponin, flavonoids in turmeric 18,19. Also, tannin, flavonoid, and saponin present in turmeric are antioxidants. Flavonoids are antioxidant, anti-allergic, anti-inflammatory, and health-promoting while Saponin has anti-inflammatory, cholesterol-lowering, and anti-fungal effects. The volatile bioactive components in turmeric can be functionalized onto smoke-derived carbon nanodots to mimic turmeric smoke's antibacterial properties. More specific investigations will be neeed to arrive at conclusive facts on the role of carbon nanodots standalone or combined with the bioactive components, that are leading to the antibacterial property.

Our group has already demonstrated the preparation of coatings on glass using separated carbon nanodots from turmeric smoke 11. The process by which the carbon coating self-assembles is explained as follows: (a) carbon particles are obtained from burning turmeric rhizomes, the soot is then collected in water, where the hydrophobic essential oils in the turmeric soot preferentially bond with the carbon particles; (b) this results in the carbon particles becoming hydrophobic as well, causing them to repel water and adhere to walls of the container; and (c) this behavior is what also drives the carbon particles to adhere to the hydrophilic glass surface when placed in the soot suspension. Using a simple dip and dry procedure, an affordable antibiofilm coating may be created. This straightforward and feasible study supports the use of carbon nanodots derived from turmeric smoke in the future to build antibiofilm coatings for electronic equipment since staphylococcal biofilms have forced medical implants to be changed too quickly.

The medical advantages of medicinal smoke cannot be obtained due to smoke's detrimental properties. However, if smoke-derived carbon nanomaterial can be extracted then they will be able to deliver the smoke effect devoid of the smoke itself. This study is only the beginning; more, targeted research in this field might produce very beneficial antibacterial substrates that are accessible and inexpensive, with uses in industry and medicine (turmeric smoke).

## Conclusions

The results indicated that the nanomaterial extracted from turmeric smoke could result in antibacterial activity against pathogenic coliforms, *E. faecalis* and *E. coli*. The carbon nanodots were also effective against the growth of oral bacteria and Staphylococcus biofilms. More in-depth studies are required to understand the mechanism of antibacterial activity.
